# Mmi1, the Yeast Ortholog of Mammalian Translationally Controlled Tumor Protein (TCTP), Negatively Affects Rapamycin-Induced Autophagy in Post-Diauxic Growth Phase

**DOI:** 10.3390/cells9010138

**Published:** 2020-01-07

**Authors:** Jana Vojtova, Jiri Hasek

**Affiliations:** Institute of Microbiology of the Czech Academy of Sciences, Videnska 1083, Prague 4 142 20, Czech Republic

**Keywords:** Mmi1, TCTP, translationally controlled tumor protein, autophagy, reactive oxygen species, rapamycin, nitrogen starvation

## Abstract

Translationally controlled tumor protein (TCTP) is a multifunctional and highly conserved protein from yeast to humans. Recently, its role in non-selective autophagy has been reported with controversial results in mammalian and human cells. Herein we examine the effect of Mmi1, the yeast ortholog of TCTP, on non-selective autophagy in budding yeast *Saccharomyces cerevisiae*, a well-established model system to monitor autophagy. We induced autophagy by nitrogen starvation or rapamycin addition and measured autophagy by using the Pho8Δ60 and GFP-Atg8 processing assays in WT, *mmi1Δ*, and in autophagy-deficient strains *atg8Δ* or *atg1Δ*. Our results demonstrate that Mmi1 does not affect basal or nitrogen starvation-induced autophagy. However, an increased rapamycin-induced autophagy is detected in *mmi1Δ* strain when the cells enter the post-diauxic growth phase, and this phenotype can be rescued by inserted wild-type *MMI1* gene. Further, the *mmi1Δ* cells exhibit significantly lower amounts of reactive oxygen species (ROS) in the post-diauxic growth phase compared to WT cells. In summary, our study suggests that Mmi1 negatively affects rapamycin-induced autophagy in the post-diauxic growth phase and supports the role of Mmi1/TCTP as a negative autophagy regulator in eukaryotic cells.

## 1. Introduction

TCTP (Translationally Controlled Tumor Protein) is an evolutionarily-conserved and abundant protein among eukaryotic organisms. It is an essential protein for the development of multicellular organisms [1,2,3] and its main biological role is likely an anti-apoptotic activity [4,5,6,7]. However, it is also involved in many other core cell biological processes (reviewed in [8]). Despite it being a long time since its discovery in the 1980s [9] and subsequent intensive studies, the protein still remains a bit enigmatic, and new discoveries and new effects are still being described. 

Recently, TCTP has also been found to affect autophagy [10,11]. Chen and colleagues reported that TCTP positively affects hypoxia and starvation-induced bulk non-selective autophagy [10]. On the other hand, Bae and colleagues have declared TCTP a negative regulator of basal and rapamycin-induced non-selective autophagy [11]. Since TCTP is an evolutionarily highly conserved protein, we used a pioneer model organism for studying autophagy, budding yeast *Saccharomyces cerevisiae* [12], reviewed in [13,14,15] to test the effect of yeast TCTP on autophagy. The *S. cerevisiae* is usually batch cultured and its growth in the culture is highly affected by a carbon source. When glucose is added to yeast cells, they rapidly adapt to fermentation of the rich carbon source during a short lag-phase. After the adaptation, they start to ferment the sugar and reach a maximal growth rate. This phase is called the exponential growth phase. Once glucose becomes limiting, yeast cells enter a second lag-phase, known as a diauxic shift [16]. During the diauxic shift yeast cells change their metabolism from fermentation to respiration. The diauxic shift is followed by a slow growing phase (post diauxic growth phase), during which ethanol, acetate, and other fermentation products are utilized by respiration. When the all carbon sources are exhausted yeast cells enter a quiescence or stationary phase (G_0_) [16,17].

Yeast TCTP was originally described as a translation machinery associated protein, Tma19 [18]. Later, Tma19 was described as a microtubule and mitochondria interacting protein and renamed Mmi1 [19]. Therefore, we refer to the protein as Mmi1 hereafter. Mmi1 is a small 18.7 kDa, acidic (pI = 4.17), and highly abundant protein in exponentially growing yeast cells corresponding to approximately 200,000 molecules per cell [20]. During the post-diauxic growth phase, Mmi1 is still a highly abundant protein exhibiting a steady-state level of expression [21], and its abundance continually decreases in the stationary phase [21,22]. Upon rapamycin treatment, the Mmi1 protein pool decreases [23], indicating that the Mmi1 expression in yeast might be regulated by TOR pathway similarly to higher eukaryotic cells [24]. Further, the *mmi1Δ* strain exhibits a slow growth phenotype [19], indicating that Mmi1 is a pro-survival factor. Mmi1 is uniformly distributed in cytosol, but if stress is applied, its distribution is changed. Upon mild oxidative stress, Mmi1 translocates to mitochondria [25], while upon heat stress it relocalizes to the nucleus and mitochondria and is also present in stress granules [26]. Mmi1 role in the nucleus is not clarified yet. However, recently Bischof and colleagues suggested a model that the mitochondrial localization of the Mmi1/TCTP is responsible for the clearance of the mitochondrial membrane from harmful proteins in a time of stress [25], thereby protecting cells from apoptosis. Above the anti-apoptotic function, Mmi1 affects a wide range of biological functions and processes most likely through interaction with its binding partners. According to the BioGRID database [27], Mmi1 currently possess about 49 physically interacting protein partners. These proteins are mainly involved in cell cycle, transcription, translation, and protein degradation. Indeed, our previous results indicated that Mmi1 modulates activity of proteasomes [26], the major protein degradation system in all eukaryotic cells next to autophagy. Nevertheless, the effect of Mmi1 on autophagy in yeast cells has not been tested yet. To test the question of whether Mmi1 affects non-selective autophagy, we induced autophagy through different conditions and used independent approaches to monitor the autophagy.

Autophagy (here referred to macroautophagy) occurs constitutively at basal levels, but it is dramatically stimulated by starvation and by various stresses [28,29]. It allows cells to respond to various types of stresses and to adapt to changing nutrient conditions [30]. Autophagy can be either a non-selective self-consumption or a selective consumption of specific cargoes or organelles. The bulk autophagy is completely inhibited in nutrient-rich conditions, but can be induced by shifting cells to starvation medium [31] or by addition of rapamycin [32], a potent inhibitor of TORC1 (target of rapamycin complex 1) [33,34]. During non-selective autophagy a portion of cytosol is sequestered for degradation into double-membrane structures named autophagosomes, which are delivered to the vacuole and degradated by vacuolar hydrolases [35]. In *S. cerevisiae*, eighteen Atg proteins, Atg1–10, Atg12–14, Atg16–18, Atg29 and Atg31 play essential roles in autophagy, and these core proteins are required for the formation of autophagosomes (reviewed in [15,36]). When non-selective autophagy is induced, the Atg17-Atg29-Atg31 complex act as an essential scaffold that facilitates formation of the preautosomal structure (PAS), from which the autophagosome is generated [37]. Autophagy is involved in a variety of physiological processes. In unicellular eukaryotes it takes care of cellular housekeeping and sustaining viability, and it is also essential for adaptation to a new host and formation of spores [38]. In higher eukaryotes it is important for cell survival and maintenance, and its dysfunction contributes to the pathologies of many diseases, e.g., cancer [39].

Here, we examined the effect of Mmi1 on bulk non-selective autophagy in yeast. Our results demonstrate a negative effect of Mmi1 on rapamycin-induced autophagy in contrast to nitrogen starvation-induced autophagy. Interestingly, the negative effect of Mmi1 on rapamycin-induced autophagy is detected after diauxic shift.

## 2. Materials and Methods

### 2.1. Yeast Strains, Media, and Growth Conditions

Yeast strains used in this study are listed in Table 1. Yeast cells were grown in shaking flask at 30 °C in rich medium (YPD; 1% *w*/*v* yeast extract, 2% *w*/*v* peptone, 2% *w*/*v* glucose) or defined synthetic medium (SD; 0.17% *w*/*v* yeast nitrogen base, 0.5% *w*/*v* ammonium sulfate, 2% *w*/*v* glucose, and auxotrophic amino acids as required). Starvation experiments were performed in synthetic minimal medium lacking nitrogen (SD-N; 0.17% *w*/*v* yeast nitrogen base without ammonium sulfate and amino acids, 2% *w*/*v* glucose).

### 2.2. Plasmids

The pAG32-*MMI1* plasmid was constructed by inserting a fragment containing 348 bps upstream of the *MMI1* ORF, *MMI1* ORF, and 454 bps downstream of *MMI1* ORF into the SacI and SpeI sites of the pAG32 plasmid. The fragment was amplified by PCR from pRS316-*MMI1* plasmid. Before the transformation of yeast cells, the plasmid was cut with BsaAI restriction enzyme at the position of 319 bps downstream of *MMI1* ORF.

The pRS316-*MMI1* plasmid was made through PCR cloning. The DNA fragment containing 500 base pairs (bps) upstream of the start codon together with the *MMI1* ORF and 500 bps downstream of the ORF were amplified by PCR from WT yeast genomic DNA. The fragment was then ligated into the SacI and EcoRI sites of pRS316 vector, resulting in the pRS316-*MMI1* plasmid.

### 2.3. Phosphatase Assay

Pho8Δ60-expressing strains (WT, *mmi1Δ* and *atg8Δ*) were grown in YPD medium to early log phase (OD_600_ ≈ 0.8) and then shifted to SD-N medium (after double wash with H_2_O and one wash with SD-N) or rapamycin was added to final concentration 200 nM. The cells were collected at indicated time points, and the alkaline phosphatase activity of Pho8Δ60 was carried out as described previously [41,43,44]. In total, five OD_600_ equivalents of yeast cells were harvested, washed once with ice-cold water, and once with wash buffer (0.85% *w*/*v* NaCl and 1mM PMSF) and resuspended in 500 μL lysis buffer (20 mM Pipes, pH 7.0, 0.5% *v*/*v* Triton X-100, 50 mM KCl, 100 mM potassium acetate, 10 mM MgSO4, 10 μM ZnSO4, and 1 mM PMSF). The cells were lysed with 250 μL equivalents of glass beads using a Fast-prep desintegrator 5 times for 20 s at 4 °C and incubated for 2 min on ice in-between. The lysates were centrifuged at 14,000× *g* for 5 min at 4 °C. The supernatant was collected and 100 μL of the supernatant was added to 400 μL reaction buffer (250 mM Tris-HCl, pH 8.5, 0.4% *v*/*v* Triton X-100, 10 mM MgSO4, and 1.25 mM p-nitrophenyl phosphate). Samples were incubated for 10 min at 30 °C before terminating the reaction by adding 500 μL of stop buffer (2 M glycine, pH 11). Production of p–nitrophenol was monitored by measuring the absorbance at 400 nm (A_400_) using a spectrophotometer (Helios Gamma Spectrophotometer, Unicam), and the concentration in nmol of p–nitrophenol in the samples was calculated from a standard curve of commercial p–nitrophenol (Sigma) (0 to 100 nmol). Protein concentration in the extracts was measured with the Pierce^TM^ BCA Protein Assay Kit (Thermo Scientific, Rockford, IL, USA), and the specific activity was calculated as nmol p–nitrophenol/min/mg protein. The statistical evaluation of phosphatase activity was performed by two-way analysis of variance (ANOVA) using R software. 

### 2.4. Quantification of Glucose

Yeast cultures were centrifuged, supernatants were collected, and glucose concentration was measured by GLU 500 kit (Erba Lachema s.r.o., Brno, Czech Republic) according to the manufactured conditions.

### 2.5. GFP-Atg8 Processing Assay

BY4741 WT, *mmi1∆*, and *atg1∆* strains transformed with plasmid pRS316_GFPAUT7 [45] carrying GFP-Atg8 were grown in SC medium to OD_600_ ≈ 0.8 and then shifted to SD-N medium (after double wash with H_2_O and one wash with SD-N) or rapamycin was added to final concentration 200 nM. In indicated time points, samples were collected and normalized to OD_600_ ≈ 1 and 100% TCA was added to the final concentration of 12.5%. Samples were frozen at −80 °C for at least 1 h, centrifuged for 5 min at 15,000× *g*, and pellets were washed with an ice-cold 80% aceton and dried at room temperature. Dried pellets were resuspended in 50 µL of 0.1N NaOH and 1% *w*/*v* SDS and bath sonicated. Then 50 µL of 2 × SDS loading buffer (100 mM Tris-HCl pH 6.8, 4% *w*/*v* SDS, 20% *v*/*v* glycerol, 0.2% *w*/*v* bromphenol blue) with 0.1M DDT was added and the lysates were resolved by 10% SDS-PAGE and transferred to Protran nitrocellulose membrane (Sigma-Aldrich/Merck, St. Louis, MO, USA). The membrane was blocked with 5% *w*/*v* non-fat dried milk (Regilait, Macon, France) and incubated overnight with a mouse monoclonal anti-GFP antibody conjugated with horseradish peroxidase (sc-9996, Santa Cruz, USA) at 1:1000. As a loading control, detection of Pgk1 with anti-Pgk1 antibody (Abcam, ab113687, 1:10000) and goat anti-mouse IgG conjugated with horseradish peroxidase (Thermo Fisher Scientific, No: 32430, 1:1000) was used. Proteins were detected by SuperSignal^TM^ West Dura Substrate (Thermo Scientific, Rockford, IL, USA). In order to calculate the ratio of free GFP to Pgk1, Western blot signals were detected by G:BOX Chemi-XRQ gel documentary system (Syngene, Cambridge, UK) and quantified by ImageJ program [46].

### 2.6. Fluorescence Microscopy

Fluorescence microscopy was performed using an Olympus IX-81 inverted microscope equipped with a motorized stage, a 100× PlanApochromat oil-immersion objective (NA = 1.4) and a Hamamatsu Orca-ER-1394 digital camera. Images were processed using Olympus Cell-R™ Xcellence, Adobe CS5 software packages. The same exposure time was used to detect GFP signal in all tested strains. To quantify the cellular distribution of GFP-Atg8 signal, cells were co-stained with a vacuolar marker FM4-64 (1 µg/mL, 1 h, 30 °C) and scored into three categories: vacuole & cytosol, cytosol, and vacuole. In the category “vacuole and cytosol” the GFP signal was evenly distributed within the cells and there were no signal gradients between vacuole and cytosol. In the category “cytosol” the higher GFP signal was detected in cytosol compared to vacuole. Finally, in the category “vacuole” the higher GFP signal was detected in vacuole compared to cytosol. The cells with oversaturated signal were excluded from the quantification.

### 2.7. Oxygen Radicals’ Measurement by Dihydroethidium Staining

Dihydroethidium (DHE) staining was performed as described in Neklesa and Davis, 2008 [47]. In total 1 × 10^7^ cells/mL were stained with 15 μg/mL of dihydroethidium (DHE) in YPD for 1 h at 30 °C under shacking. Cells were washed in PBS, reinoculated in PBS, and in total 20,000 cells were analyzed in a Flow Cytometer BD LSRII (BD Biosciences, USA). To detect specifically the DHE oxidation product hydroxyethidium, an excitation wavelength of 405 nm was used. This wavelength is the closest available wavelength to a distinct hydroxyethidium excitation maximum of 396 nm that is not present for other DHE oxidation products [48]. The signal was collected by using emission filter 576/26, and cell viability was evaluated by Hoechst 33,258 (1 µg/mL). The data were analyzed by FlowJo software and mean fluorescence DHE intensity values were used to compare the total DHE fluorescence of the strains. 

### 2.8. Viability Assay for Testing Rapamycin Sensitivity

To compare the sensitivity of WT and *mmi1∆* strains to rapamycin, we incubated exponentially growing cells in 24 well plate in YPD with 0, 5, 6, 8, 10, and 15 nM of rapamycin. The cells were seeded to initial OD ≈ 0.1 and incubated overnight at 30 °C in Eon^TM^ Miroplate Spectrophotometer (BioTek) under agitation. Every 15 min OD_600_ was determined in each well by Gen5 Microplate Reader and Imager Software. Due to increased concentration of rapamycin were obtained outgrowth curves with a distinct shift in the curves as cells lose viability. For each concentration time shift for OD ≈ 0.5 in the outgrowth curve relative to the curve of untreated cells was determined and viability curves were calculated as described in details in [49].

## 3. Results

### 3.1. Basal and Nitrogen Starvation-Induced Autophagy are not Affected by Mmi1

**Figure 1:**
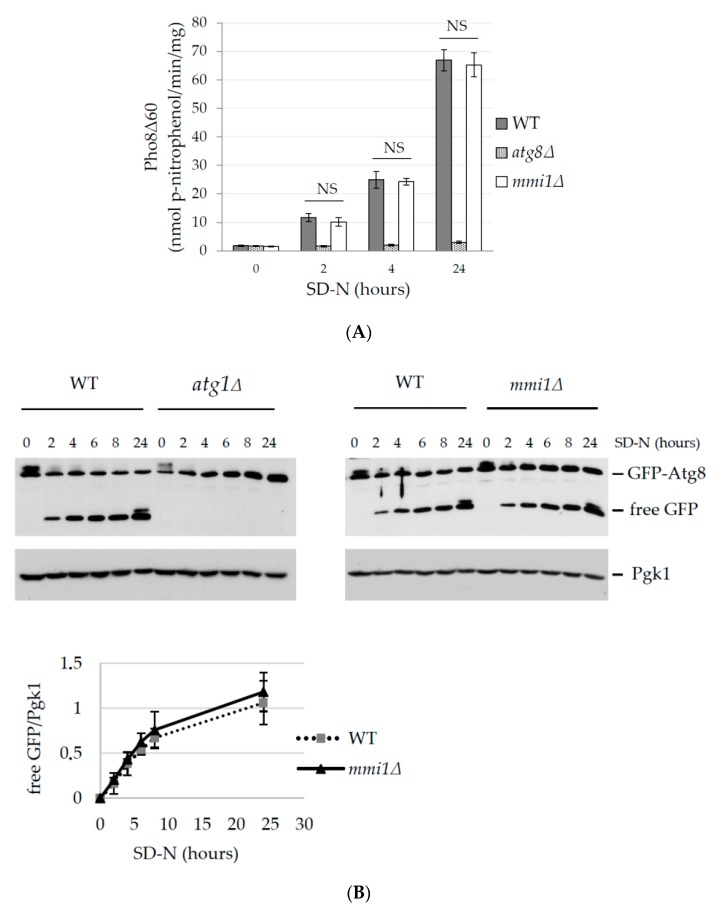
Basal and nitrogen starvation-induced autophagy normally occur in *mmi1Δ* cells. (**A**) Exponentially growing WT, *atg8Δ*, and *mmi1Δ* cells expressing Pho8Δ60 (OD_600_ ≈ 0.8) were shifted to nitrogen starvation medium (SD-N). Samples were taken in indicated time points, proteins extracted, and the specific Pho8Δ60 activity was determined. Results are means ± SD of three independent experiments performed in duplicates (n = 6). The statistical evaluation was performed by using two way analysis of variance (ANOVA). The threshold for significance was set as *p* ≤ 0.01. NS; not significant; (**B**) Western blot detection of GFP-Atg8 cleavage in the WT, *atg1Δ*, and *mmi1Δ* cells. Cells expressing GFP-Atg8 were grown until the logarithmic growth phase (OD_600_ ≈ 0.8) and then shifted to the SD-N medium. Samples were taken at indicated time points and the cleavage of GFP-Atg8 was analyzed by Western blot detection with antibodies against GFP. Detection of Pgk1 was used as a loading control. Quantification of the blots is presented below. GFP ratio (free GFP/Pgk1) was calculated. Error bars reflect SD from the two independent experiments.

A previous study in mammalian cells indicated that TCTP promotes autophagy under hypoxia and starvation conditions [10]. Later, however, the results were challenged by a study demonstrating TCTP as a negative regulator of non-selective autophagy [11]. To investigate the role of the yeast TCTP ortholog, Mmi1, in non-selective autophagy, we examined autophagy in wild-type (WT) and *mmi1Δ* cells upon shift to a nitrogen starvation medium that stimulates autophagy induction [12]. As a negative control we used autophagy deficient strains *atg8Δ* depleted for the key autophagy molecule Atg8 [50]. The non-selective autophagy was determined by the quantitative Pho8Δ60 assay [43]. The Pho8Δ60 assay is an enzymatic assay that utilizes a truncated version of the alkaline phosphatase Pho8Δ60 that lacks the targeting sequence to endoplasmic reticulum, and thus remains in the cytosol. Upon autophagy induction, Pho8Δ60 is delivered to the vacuole, gets activated by the proteolytic cleavage, and serves as a marker of the amount of cytosol delivered through the non-selective autophagy [43]. Yeast strains were grown in the YPD medium until the early logarithmic phase, washed, and shifted to the nitrogen starvation (SD-N) medium. As shown in Figure 1A, at 0 h upon shift to SD-N media low values of the phosphatase activity were detected in all tested strains, indicating that basal levels of autophagy were not affected. Upon prolonged nitrogen starvation, phosphatase activities increased. However, similar values were detected for the WT and the *mmi1Δ* strains at all tested time points. On the other hand, the control *atg8Δ* strain exhibited only a low level of autophagy, demonstrating that delivery of Pho8Δ60 to the vacuole depends on autophagy. These results indicated that Mmi1 did not affect the nitrogen starvation-induced non-selective autophagy. 

To confirm the results by an independent approach we also performed GFP-Atg8 processing assay [28]. The GFP-Atg8 assay is based on Western blot detection of a free GFP moiety released from a core autophagy protein Atg8 that is resistant to vacuolar proteases [28]. As a negative control we used autophagy deficient *atg1Δ* depleted for the key autophagy molecule Atg1 [51]. As shown in Figure 1B, no free GFP band was detected by Western blot at 0 h time point of nitrogen starvation in WT, *mmi1Δ*, and *atg1Δ* strain demonstrating that basal level of autophagy was not affected in the strains. Upon prolonged nitrogen starvation, similar levels of the free GFP band were detected in the WT and the *mmi1Δ* cell lysates, indicating that Mmi1 did not influence GFP-Atg8 cleavage upon nitrogen starvation. Further, no free GFP band was detected in the negative control *atg1Δ* strain at all tested time points, demonstrating that GFP-Atg8 cleavage was dependent on the autophagic degradation. These results, together with the results obtained by the phosphatase assay, demonstrate that the basal and the nitrogen starvation-induced autophagy are not influenced by Mmi1 in yeast *S. cerevisiae*.

### 3.2. Mmi1 Negatively Affects Rapamycin-Induced Autophagy When the Cells Enter Post-Diauxic Growth Phase

**Figure 2:**
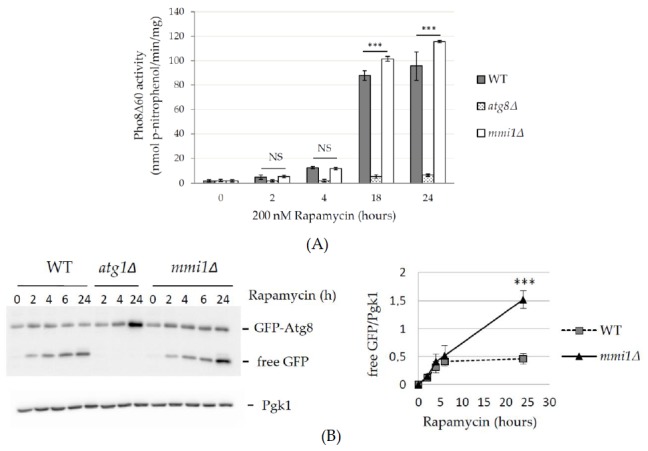
Non-selective autophagy is promoted in the *mmi1Δ* strain after prolonged incubation with rapamycin. (**A**) Exponentially growing WT, *atg8∆*, and *mmi1∆* cells expressing Pho8Δ60 (OD_600_ ≈ 0.8) were treated with rapamycin 200 nM. Samples were taken at indicated time points, proteins extracted, and the specific Pho8∆60 activity was determined. Results are means ± SD of two independent experiments performed in duplicates (n = 4). The statistical evaluation was performed by using two way analysis of variance (ANOVA). NS; not significant; *** *p* < 0.001. (**B**) Western blot detection of GFP-Atg8 cleavage in WT, *atg1∆*, and *mmi1∆* cells after rapamycin addition. Cells expressing GFP-Atg8 were grown until log phase (OD ≈ 0.8) and then rapamycin was added to a final concentration 200 nM. At indicated time points proteins were extracted and the protein lysates were examined by Western blot. Pgk1 was used as a loading control. Quantification of the blots is presented to the right. GFP ratio (free GFP/Pgk1) was calculated. *** *p* < 0.01. Error bars reflects SD from three independent experiments.

A previous study on HeLa cells demonstrated a negative effect of TCTP on rapamycin-induced non-selective autophagy [11]. Therefore, we also examined the effect of Mmi1 on autophagy induced by rapamycin, a potent inhibitor of the TOR pathway [33,34] that stimulates autophagy [32]. Yeast cells were grown in rich YPD medium until the early logarithmic growth phase. Autophagy was induced by the addition of rapamycin and determined by the phosphatase assay. As shown in Figure 2A, very low autophagy levels were detected at 0 h after rapamycin addition in all strains, demonstrating consistently with our previous results that the basal autophagy is not affected by Mmi1. After prolonged incubation with rapamycin, autophagy levels increased but no difference between the WT and the *mmi1∆* strains was detected 2 and 4 h after the autophagy induction. However, an increased autophagy was detected 18 and 24 h after rapamycin addition to *mmi1∆* cells (Figure 2A). Low levels of autophagy were detected in the control *atg8∆* cells in all tested time points. These results indicated that rapamycin-induced autophagy was increased in the *mmi1Δ* strain after a long term incubation with rapamycin.

**Figure 3:**
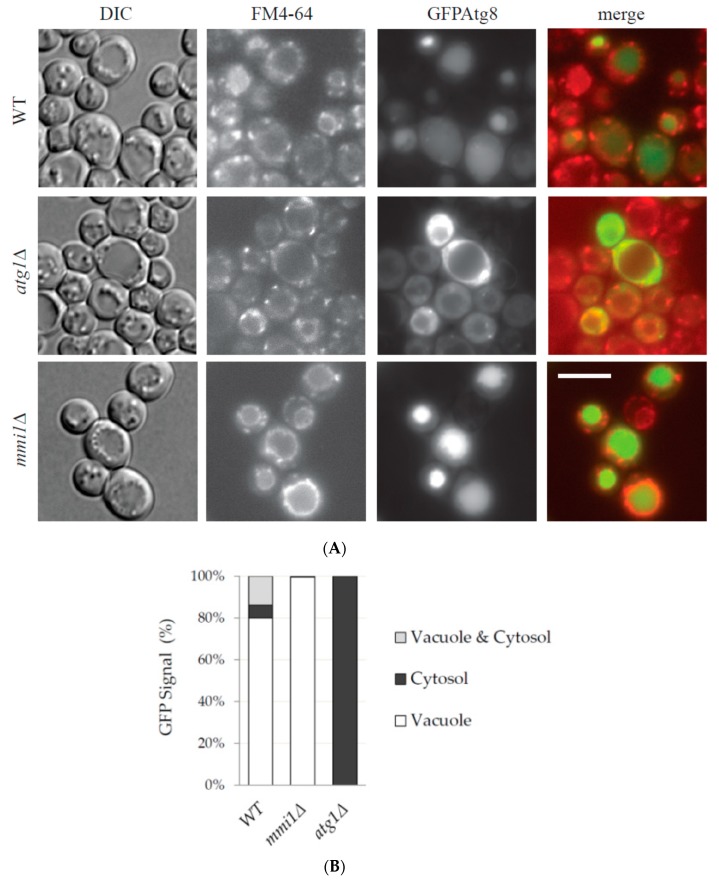
Fluorescence microscopy detection of a higher GFP-Atg8 processing in *mmi1∆* cells upon rapamycin treatment. (**A**) The same samples of WT, *atg1∆*, and *mmi1∆* cells treated with rapamycin for 24 h and analyzed by Western blot in Figure 2B were examined under fluorescence microscope. Vacuoles were labelled by the red fluorescence probe FM4-64 (1 µg/mL, 1h) and GFP signal was taken under the same exposure time in all tested strains. DIC, differential interference contrast. Scale bar, 5 µm. (**B**) Quantification of the GFP-Atg8 cellular distribution 24 h after rapamycin addition in WT, *mmi1∆*, and *atg1∆* strains. Results are means from two independent experiments and each bar represents 250 cells.

To corroborate the results, we also performed GFP-Atg8 processing assays. Consistently, similar levels of the free GFP were detected in the WT and *mmi1Δ* cell lysates at early time points upon autophagy induction, and no free GFP was detected in the control *atg1Δ* cell lysates at all tested time points (Figure 2B). However, an increased accumulation of the free GFP was detected in the *mmi1Δ* cell lysate 24 hours after rapamycin addition. The increased GFP-Atg8 processing in *mmi1Δ* strain 24 hours upon rapamycin addition could be also confirmed by fluorescence microscopy (Figure 3). The majority of the GFP-Atg8 signal in the *mmi1Δ* cells was present in the vacuole compared to WT cells, while in the *atg1Δ* cells the majority of the GFP signal was present outside of the vacuole (Figure 3A, B). These results, together with the results of the phosphatase assay demonstrate the increased autophagy in the *mmi1Δ* strain at later time points after rapamycin addition.

**Figure 4:**
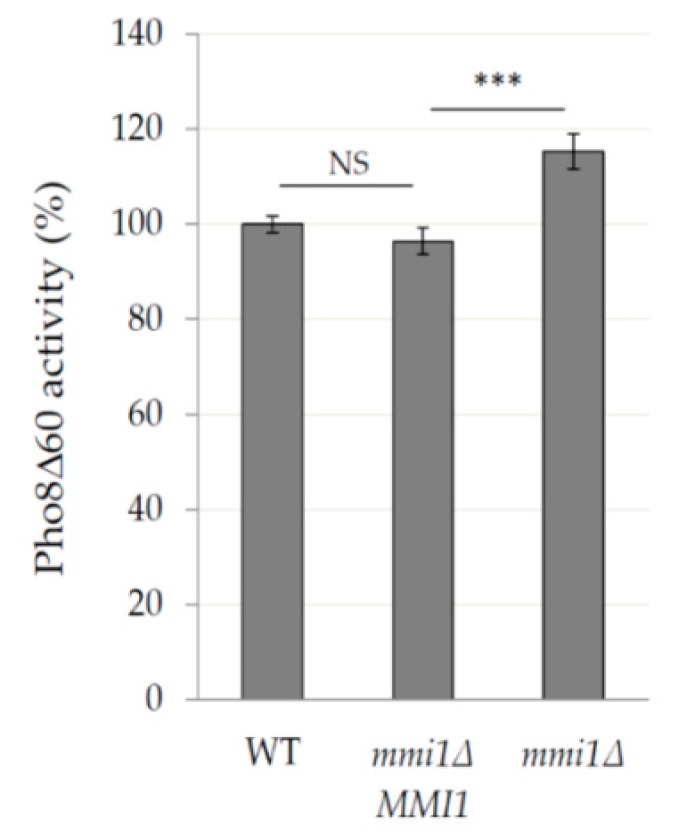
The increased rapamycin-induced autophagy in the *mmi1∆* strain can be rescued by wild-type *MMI1* gene. Exponentially growing cells of the WT, *mmi1∆*, and *mmi1∆ MMI1* strains expressing Pho8Δ60 (OD_600_ ≈ 0.8) were treated with 200 nM rapamycin for 24 h and the specific Pho8*∆*60 was determined. Results are expressed as means ± SD from two independent experiments performed in duplicates (n = 4). The statistical evaluation was performed by using two way analysis of variance (ANOVA). *** *p* < 0.001; NS; not significant.

To verify that the phenotype seen in the *mmi1∆* mutant strain was not due to an unknown secondary mutation, we created a new strain (CRY2959) possessing the wild-type *MMI1* gene under control of its endogenous promotor in the *mmi1∆* strain. As shown in Figure 4, the presence of *MMI1* wild-type gene decreased the autophagy activity of *mmi1∆* strain to the WT strain, suggesting that the mutant phenotype is indeed the result of deleting the *MMI1* gene.

**Figure 5:**
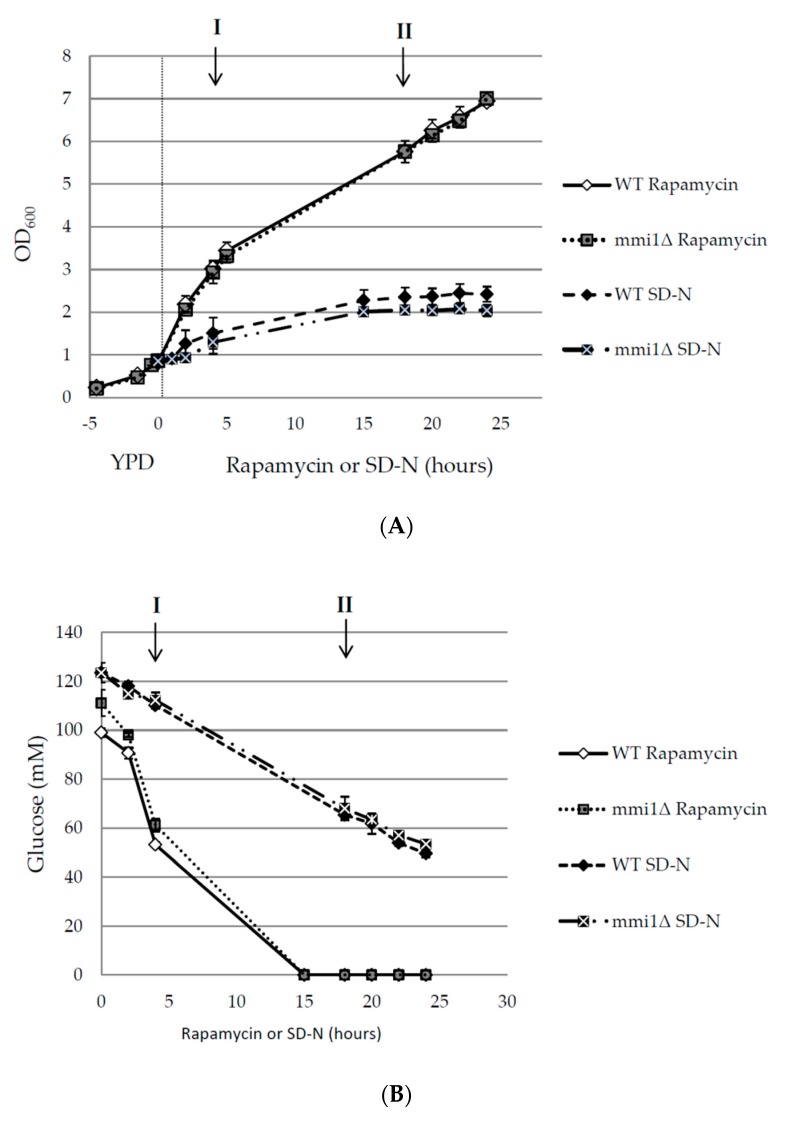

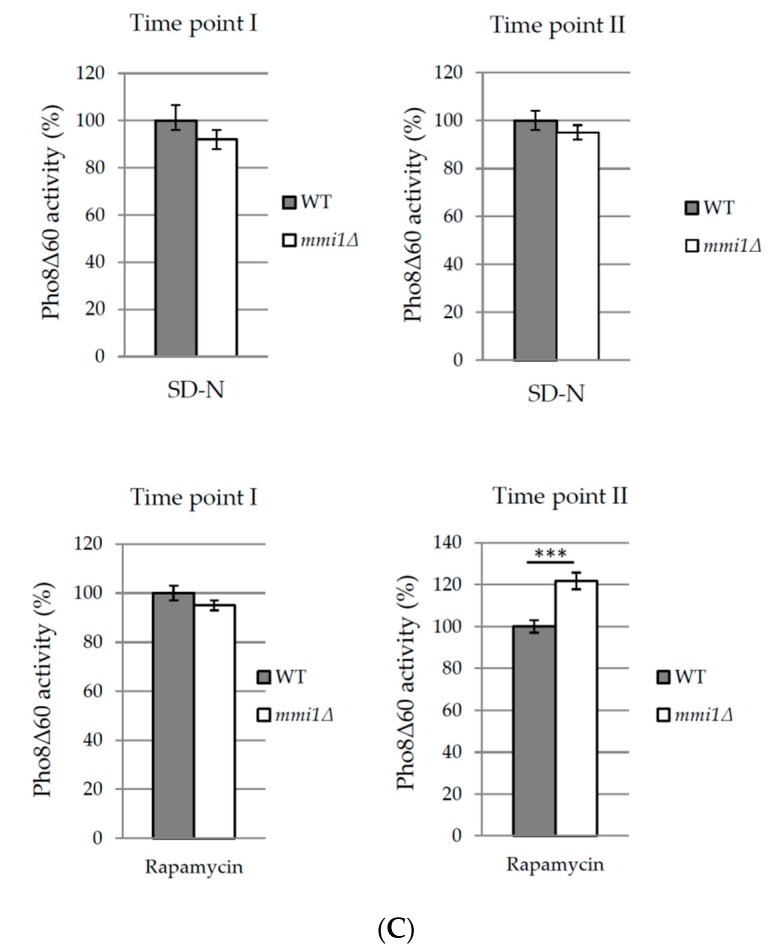
Increased rapamycin-induced autophagy in *mmi1Δ* strain occurs after glucose exhaustion. (**A**) Exponentially growing WT and *mmi1∆* cells expressing Pho8Δ60 (OD_600_ ≈ 0.8) in the YPD medium were either shifted to the SD-N medium or treated with rapamycin (200 nM). Optical density at 600 nm was measured at indicated time points. Results are presented as means ± SD of three independent experiments performed in duplicates (n = 6). (**B**) Concentration of glucose in the media as shown in A was measured after the addition of rapamycin (200 nM) to WT and *mmi1∆* strains or after the shift of the strains to SD-N media. Results are means of two independent experiments performed in triplicates (n = 6). (**C**) Pho8Δ60 assay was measured in time points I and II as indicated in A, and B. Point I represents the exponential growth phase where glucose is still present in the medium. Point II represents the post-diauxic phase where glucose is already exhausted. Results are normalized to the WT strain (100%) and represent means ± SD from two independent experiments performed in duplicates (n = 4). The statistical evaluation was performed by using two way analysis of variance (ANOVA). *** *p* < 0.001.

Our results demonstrated the increased autophagy in *mmi1∆* cells after a long-term incubation with rapamycin. However, no similar effect was detected in the case of the nitrogen starvation-induced autophagy. Nitrogen starvation and rapamycin treatment are widely used approaches to induce autophagy in yeast [31,32]. In the nitrogen starvation conditions, yeast cells complete division and arrest in the G1/G2 quiescence state [52], increase their volume due to enlarged vacuole by autophagy induction, and remain viable for two days [12]. Rapamycin treatment is believed to mimic nutrient deprivation, including greatly reduced cell growth [33] and autophagy induction [32]. However, it has been demonstrated independently by several groups that even at high concentration of rapamycin, yeast cells maintain their proliferative ability [47,53,54,55,56]. We used batch cultivation, and cells were grown in rich YPD media to OD_600_ ≈ 0.8 before rapamycin was added to a final concentration 200 nM, or cells were washed and shifted to nitrogen starvation media. Both approaches induced autophagy as shown earlier (see Figure 1 and Figure 2). Nevertheless, as shown in Figure 5, rapamycin and nitrogen starvation-treated cells highly differed in the cell growth. In nitrogen starvation medium the WT and the *mmi1∆* strains reached only OD_600_ ≈ 2. In fact, the OD_600_ value in the SD-N medium seems to be overestimated likely due to an increased size of the cells. Indeed, when the number of cells per ml was measured, the increase of about 20% only was detected (Supplementary Figure S1). On the other hand, WT and *mmi1∆* strain treated with 200 nM rapamycin exhibited higher growth rates and the cell cultures reached OD_600_ ≈ 7 after 24 h of cultivation after rapamycin addition. These results demonstrate a striking difference between used nitrogen starvation conditions and rapamycin treatment in batch-cultivated yeast cells. To characterize the cell growth during nitrogen starvation and rapamycin treatment, we measured concentration of glucose in media. As showed in Figure 5B, glucose was still present even 24 h upon the shift to the SD-N media. However, glucose was completely exhausted in the YPD media at latest 15 h after rapamycin addition (Figure 5B). Since *S. cerevisiae* cultivated in the glucose-containing media undergoes a growth arrest (diauxic shift) after glucose depletion, no presence of glucose in the media demonstrates that the cells have already entered the post diauxic growth phase. To correlate glucose depletion with autophagy induction, we measured phosphatase activity in indicated time points I and II as shown in Figure 5A,B. The time point I represented an exponential phase, where glucose was present in media and the time point II represented the post-diauxic growth phase; where also glucose was absent from the media (Figure 5B). Our results indicate that autophagy is promoted in the *mmi1∆* strain after rapamycin treatment, when glucose is already not present in the media (Figure 5C). These results altogether demonstrate that the rapamycin-induced autophagy is increased in the *mmi1∆* strain after diauxic shift.

### 3.3. In Post Diauxic Growth Phase Amount of Superoxide Radicals is Decreased in mmi1∆ Strain

**Figure 6:**
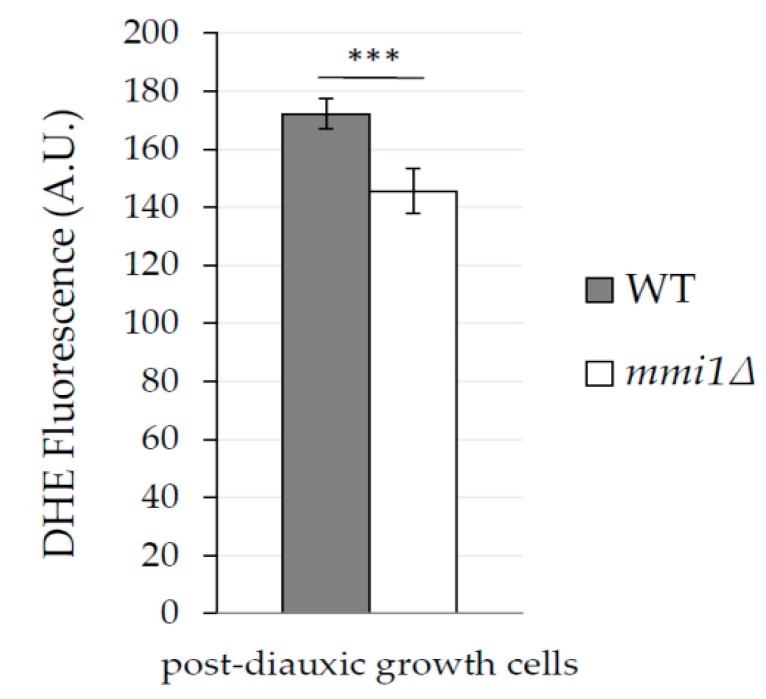
In post diauxic growth phase the *mmi1∆* strain exhibits lower amount of superoxide anions compared to the WT strain. The amount of superoxide anions was determined in the WT and *mmi1∆* strains 18 h after rapamycin treatment (200 nM). The production of superoxide anions was measured by dihydroethidium (DHE, 15 µg/mL, 1 h, 30 °C) and samples were analyzed by the FACS flow cytometer. Results are means ± SD of two independent experiments performed in triplicates (n = 6). The statistical evaluation was performed by using two way analysis of variance (ANOVA). *** *p* < 0.001.

**Figure 7:**
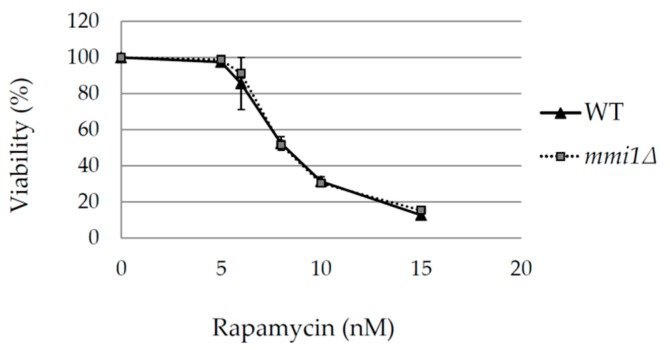
Sensitivity to rapamycin is not influenced in the *mmi1∆* strain. WT and *mmi1∆* cells were incubated in the presence of an indicated concentration of rapamycin. Survival curves for the WT and the *mmi1∆* strains were generated from outgrowth curves and represent the average viabilities of four biological replicates for each strain. Results are means ± SD of two independent experiments performed in duplicates (n = 4).

Our results demonstrated that Mmi1 negatively affects the rapamycin-induced autophagy in the post diauxic growth phase. Rapamycin forms a complex with FKBP (Fpr1 in yeast) to inhibit TORC1 [33]. Neklesa and Davis reported that superoxide anions regulate TORC1, and its ability to bind Fpr1:rapamycin complex in *S. cerevisiae* [47]. According to the authors, elevated levels of superoxide anions modify TORC1 that it is no longer able to fully bind Fpr1:rapamycin complex. To test the possibility that the increased rapamycin-induced autophagy in the *mmi1∆* strain results from a lower pool of superoxide anions, we stained the WT and the *mmi1∆* cells with dihydroethidium (DHE), a superoxide anions sensitive probe. The exponentially growing cells were treated with rapamycin for 18 h to reach the post-diauxic growth phase, then the cells were labeled with DHE, and analyzed by FACS flow cytometer. As shown in Figure 6, *mmi1∆* cells exhibited significantly decreased amount of superoxide anions compared to the WT cells. This suggested that the decreased pool of superoxide anions in *mmi1∆* cells might contribute to the stronger interaction between rapamycin/Fpr1 and TORC1 and, hence, it might promote the non-selective autophagy. To further test this hypothesis, we analyzed sensitivity of the WT and the *mmi1∆* strains to rapamycin. We assume that the *mmi1∆* strain should be more sensitive to rapamycin compared to WT strain if the decreased amount of ROS in *mmi1∆* strain facilitate binding of Fpr1:rapamycin complex to TORC1. We cultivated the WT strain and the *mmi1∆* strain in the presence of increasing concentrations of rapamycin, and calculated survival curves as described in [49]. As shown in Figure 7, the WT and the *mmi1∆* strains exhibited the same sensitivity to rapamycin. These results indicate no correlation between the decreased ROS production and sensitivity to rapamycin in the *mmi1∆* strain. This suggests that the decreased ROS production in the *mmi1∆* strain likely does not facilitate the Fpr1:rapamycin binding to TORC1. 

## 4. Discussion

As TCTP is a conserved protein from yeast to human and autophagy is a conserved protein degradation pathway, we speculated that examination of the role of yeast TCTP (Mmi1 protein) in autophagy might help us to understand controversial results from higher eukaryotic cells, indicating both positive and negative effects on autophagy [10,11]. Using batch-cultivated yeast cells of *S. cerevisiae*, we demonstrated that in exponentially growing cells Mmi1 protein had no effect on basal or nitrogen starvation-induced bulk non-selective autophagy (Figure 1). However, if exponentially growing cells were treated with rapamycin, Mmi1 negatively affected autophagy (Figure 2, Figure 3 and Figure 4) when the cells entered the post-diauxic growth phase (Figure 5). Further, in the post-diauxic growth phase we detected lower amount of superoxide radicals in *mmi1∆* cells compared to WT cells (Figure 6). Our results also indicate that WT and *mmi1∆* cells possess same sensitivity to rapamycin (Figure 7).

Previously, Chen and colleagues suggested that mammalian TCTP could positively regulate autophagy. By using African green monkey kidney fibroblast-like cells (COS-7 cells) they demonstrated that TCTP knockdown inhibits autophagy under hypoxic or serum starvation conditions [10]. The effect of hypoxic conditions on autophagy has not been tested in this study and awaits further exploration. However, when the exponentially growing yeast cells were shifted from the rich YPD medium to nitrogen starvation conditions, high autophagy levels were induced but no effect of Mmi1 was detected. These results are consistent with the finding that a very slight or no effect on autophagy was previously detected in response to nutrient starvation condition in the knockdown TCTP human HeLa cell line and mouse embryonic fibroblasts (MEFs), haploinsufficient in TCTP expression [11]. 

Further, contrary to a positive role of TCTP on autophagy, Bae and colleagues reported that TCTP/TPT1 negatively regulates autophagy [11]. They detected increased autophagy in HeLa cells transiently transfected with *TPT1* shRNA or in mouse embryonic fibroblasts (MEFs) from heterozygote knockout mice embryos (*Tpt1*^+/−^). The negative autophagy regulation was potentiated by rapamycin and an increased autophagy was also demonstrated in vivo in livers and kidneys of *Tpt1* heterozygote mice [11]. Our results indicate that in yeast cells Mmi1 negatively regulates the rapamycin-induced autophagy (Figure 2 and Figure 3) when glucose is exhausted from media (Figure 5B) and the cells enter post-diauxic growth phase. In contrast, no effect on autophagy is detected under nitrogen starvation condition (Figure 1) when glucose is still present in the medium (Figure 5B).

It is generally accepted that both rapamycin treatment and nitrogen starvation inhibit downstream the TOR signaling pathway that results in repression of protein translation and proliferation and leads to autophagy stimulation [31,32]. However, our results indicate a different effect of Mmi1 on autophagy, based on the used conditions. The rapamycin-induced autophagy might differ from the nitrogen starvation-induced autophagy somewhere within TOR inhibition and/or downstream of TOR signaling that happens during post diauxic growth phase that is not present during nitrogen starvation.

Rapamycin has been thought to fully deactivate the budding yeast TORC1 and driving cells into a quiescent/G0 state [33]. However, this dogma has changed since many groups reported only slowed proliferation upon rapamycin treatment [47,53,54,55] and only partial inhibition of yeast TORC1 [54]. In fact, rapamycin also partially inactivates mammalian mTORC1 [57,58]. It seems evident that maintained proliferative activity upon rapamycin treatment is crucial for detection of increased autophagy induction in *mmi1∆* cells in our study. Interestingly, we have noticed that upon rapamycin addition to exponentially growing cells both, the WT and the *mmi1∆* strain grow similarly (Figure 5A), and the *mmi1∆* strain lost its slow growth phenotype [19]. Since Mmi1 possesses pro-survival activity, we might speculate that during the exponential growth Mmi1 possesses some activity that is directly regulated by TORC1.

Upon rapamycin treatment we detected the increased autophagy in *mmi1∆* cells of the post-diauxic growth phase. This phenotype could be rescued by the wild type *MMI1* gene inserted into the *mmi1∆* strain (Figure 4). At the diauxic shift cells switch from fermentation to respiration and from rapid proliferation to slow proliferation [59]. Further, PKA and TOR pathways are downregulated, and PKC and Snf1 pathways are activated, the former transiently [60], the latter important for the induction of a carbon starvation autophagy in cells undergoing the diauxic shift [61]. Importantly, the carbon starvation autophagy is not induced when the cells are grown in glucose medium, and then shifted to carbon starvation media [61]. It requires an absence of catabolite repression [61] that is responsible for a preferential utilization of glucose to other carbon sources and strict repression of respiration [62]. At the moment the mechanism how Mmi1 negatively affects the rapamycin-induced autophagy in the post-diauxic phase is unknown and awaits a detailed exploration. 

However, it has been reported that the reactive oxygen species can hamper inhibitory activity of rapamycin in *S. cerevisiae* by oxidative damage to yeast TORC1 [47]. Our results indicate that in the post-diauxic growth phase WT cells exhibit a higher amount of reactive oxidative species compared to *mmi1∆* cells (Figure 6). Since WT and *mmi1∆* cells exhibit the same sensitivity to rapamycin (Figure 7), it seems unlikely that the decreased ROS production in the *mmi1∆* strain influences rapamycin binding to TORC1. Further, the reactive oxygen species accumulation was shown to be critical for autophagy induction during nutrient starvation conditions in mammalian cells [63] and a number of studies indicate autophagy regulation by redox signaling [64]. In *S. cerevisie*, activity of cysteine protease Atg4 could be regulated by the redox state and, hence, it may regulate autophagosome formation [65]. Recently, ethanol stress-induced autophagy was reported to also be regulated by ROS [66]. 

Here, we used exponentially growing cells and induced autophagy by nitrogen starvation or rapamycin treatment. These approaches were introduced already in 1990s [31,32] and have been used by many groups to trigger autophagy in cells previously grown in the rich YPD medium. Nevertheless, in a natural habitat, cells face large variations in nutrients, and some autophagy roles in cellular metabolism seems to be still unexplored [67]. In this respect, Iwama and Ohsumi recently reported that a bulk autophagy is activated in batch culture on low glucose media based on available carbon sources [68]. Further, Horie and colleagues reported that iron recycling via autophagy is critical for transition from glycolytic to respiratory growth [67]. Several experiments are now in progress to test the effect of Mmi1 on autophagy induced during cell aging and quiescence. So far, our results support the role of Mmi1/TCTP as a negative regulator in the rapamycin-induced non-selective autophagy in eukaryotic cells.

## Figures and Tables

**Table 1:** Yeast strains used in this study.

Strain	Relevant Genotype	Source
CRY155	BY4741; *MATa, his3Δ1 leu2Δ0 met15Δ0 ura3Δ0*	[40]
CRY1107	BY4741; *MATa mmi1::KanMX4*	Euroscarf
CRY2829	*MATa, leu2–3,112, trp1, ura3–52, pho8::pho8Δ60, pho13::URA3*	[41]
CRY2830	*MATa, leu2–3,112, trp1, ura3–52, pho8::pho8Δ60, pho13::URA3, atg8::KanMX*	[42]
CRY2833	*MATa, leu2–3,112, trp1, ura3–52, pho8::pho8Δ60, pho13::URA3, mmi1::natNT2*	This study
CRY2645	BY4741; *MATa [pRS316GFPAut7]*	This study
CRY2673	BY4741; *MATa, mmi1::KanMX4 [pRS316GFPAut7]*	This study
CRY2662	BY4741; *MATa, atg1::natNT2*	This study
CRY2665	BY4741; *MATa, atg1::natNT2 [pRS316GFPAut7]*	This study
CRY2959	MATa, *leu2–3,112, trp1, ura3–52, pho8::pho8Δ60, pho13::URA3, mmi1::natNT2 pAG32-MMI1 (hphMX6)*	This study

## Supplementary Materials

The following are available online at https://www.mdpi.com/2073-4409/9/1/138/s1, Figure S1: Growth of WT and *mmi1Δ* strains in nitrogen starvation media.
