# Supplementary material for: Mmi1, the Yeast Ortholog of Mammalian Translationally Controlled Tumor Protein (TCTP), Negatively Affects Rapamycin-Induced Autophagy in Post-Diauxic Growth Phase
Source: Cells. 2020 Jan 7;9(1):138. doi: 10.3390/cells9010138 (PMC7017036; doi:10.3390/cells9010138)
Supplement: Supplementary file 1 [file cells-09-00138-s001.pdf]

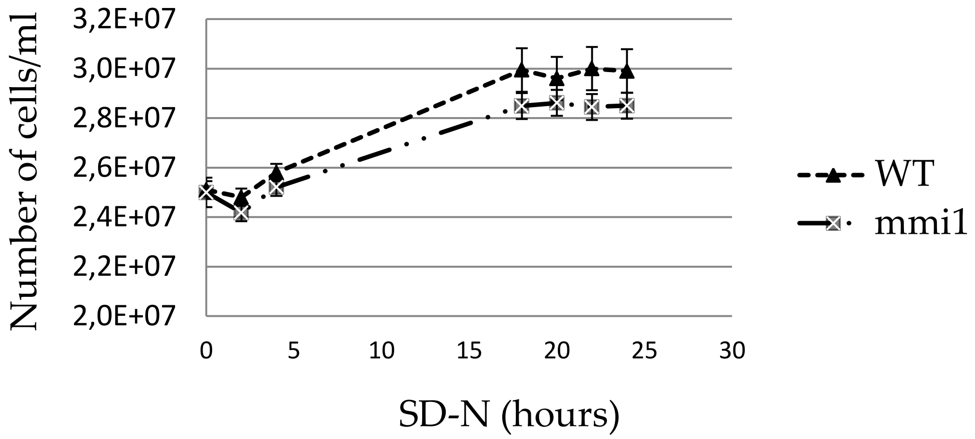

Figure S1. Growth of WT and *mmi1Δ* strains in nitrogen starvation media. Exponentially growing WT and *mmi1Δ* strain (OD ≈ 0.8) were shifted from YPD to SD-N media. Number of cells per ml was determined at indicated time points. Results are means ± SD (n=3).
